# Promoted cobalt metal catalysts suitable for the production of lower olefins from natural gas

**DOI:** 10.1038/s41467-018-08019-7

**Published:** 2019-01-11

**Authors:** Jingxiu Xie, Pasi P. Paalanen, Tom W. van Deelen, Bert M. Weckhuysen, Manuel J. Louwerse, Krijn P. de Jong

**Affiliations:** Inorganic Chemistry and Catalysis, Debye Institute for Nanomaterial Science, Utrecht University, Universiteitsweg 99, 3584 CG Utrecht, The Netherlands

## Abstract

Due to the surge of natural gas production, feedstocks for chemicals shift towards lighter hydrocarbons, particularly methane. The success of a Gas-to-Chemicals process via synthesis gas (CO and H_2_) depends on the ability of catalysts to suppress methane and carbon dioxide formation. We designed a Co/Mn/Na/S catalyst, which gives rise to negligible Water-Gas-Shift activity and a hydrocarbon product spectrum deviating from the Anderson–Schulz–Flory distribution. At 240 °C and 1 bar, it shows a C_2_-C_4_ olefins selectivity of 54%. At 10 bar, it displays 30% and 59% selectivities towards lower olefins and fuels, respectively. The spent catalyst consists of 10 nm Co nanoparticles with hcp Co metal phase. We propose a synergistic effect of Na plus S, which act as electronic promoters on the Co surface, thus improving selectivities towards lower olefins and fuels while largely reducing methane and carbon dioxide formation.

## Abstract (web summary)

The conversion of natural gas to chemicals and fuels via the Fischer-Tropsch synthesis process, depends on the ability of catalyst materials to suppress the formation of methane and carbon dioxide. Here, the authors develop a Co_1_Mn_3_-Na_2_S catalyst, which has a hydrocarbon product spectrum deviating from the Anderson–Schulz–Flory distribution.

## Introduction

The abundant availability of methane feedstock due to the shale gas revolution decreases the dependence on crude oil, however new technologies have to be developed to utilize its potential^1,2^. Methane may be converted to synthesis gas (syngas, a mixture of H_2_ and CO), which can then be used to produce chemicals and fuels via the Fischer-Tropsch synthesis (FTS) process^3^. FTS is a surface polymerization reaction so the product selectivity is governed by the Anderson–Schulz–Flory (ASF) distribution^4^. Deviation of the ASF distribution to suppress methane formation is critical to attain high fractions of lower olefins (ethylene, propylene, and butylenes), and this is possible with promoted Fe-carbide-based^5–7^ and promoted Co-carbide-based catalysts^8,9^. However, most carbide-based catalysts are also active for the water-gas-shift (WGS) reaction^10^, thereby producing CO_2_ and rendering them inefficient for methane-derived H_2_-rich syngas. Similarly, the bifunctional oxide-zeolite catalysts, which convert syngas directly to lower olefins, showed high activity for WGS and are thus only suitable for CO-rich syngas^11,12^. The importance of decreasing CO_2_ production during the FT step was recently highlighted by Wang et al. in their development of phase pure, stable and low-CO_2_ selective ε-iron carbide FT catalysts for the coal-to-liquids process^13^.

To be active for FTS but not for WGS, Co has to be in the metallic state during catalysis. Metallic Co-based catalysts are used commercially for the gas-to-liquids process in which long-chain saturated hydrocarbon products are produced that are subsequently cracked to valuable transportation fuels in particular kerosene and diesel^14–17^. The direct production of lower olefins from H_2_-rich syngas is advocated, but this poses two challenges, specifically the suppression of methane and of CO_2_ formation during FTS.

Till now, Co-based catalysts for the direct conversion of syngas to lower olefins focused on MnO as promoter, but the product spectrum was still dictated by the ASF distribution^18–22^. Adding alkali promoters to Co/MnO catalysts stimulates formation of Co-carbide, which inhibits methane, but promotes CO_2_ production^23^. Besides acting as structural promoters, alkali metals were established to decrease activity for metallic Co-based catalysts and it was proposed to be correlated to the element electronegativity^24,25^. These alkali metals including Na or K, exist as oxides Na_2_O or K_2_O during catalysis, yet the oxygen counter-ion was often overlooked. The importance of counter-ions to alkali metal promoters was demonstrated previously for Fe-based catalysts^26^, particularly the combination of Na and S was found to give a synergistic effect^27–29^. S is generally perceived to be a poison for Co-based catalysts in terms of activity and selectivity towards long-chain hydrocarbons (C_5+_)^30^, however it was also shown to decrease chain growth probability and improve olefins selectivity depending on its concentration^31–33^. Nonetheless, the influence of alkali metal and its counter-ion has not been considered for Co-based catalysts.

In this work, we demonstrate that the presence of Na plus S inhibits WGS and suppresses methane formation for metallic Co-based catalysts. We present an efficient metallic Co-based catalyst consisting of Co/Mn/Na/S, which has a product spectrum deviating from ASF distribution yet is inactive for WGS. The catalytic performance of this catalyst is evaluated over a range of reaction temperatures, 240–280 °C, and reaction pressures, 1–10 bar. H_2_/CO feed ratio is kept constant at 2, a stoichiometric ratio relevant for methane feedstock. At industrially relevant conditions of 240 °C and 10 bar, Co_1_Mn_3_–Na_2_S shows superior product selectivities towards lower olefins and fuels in comparison to other Co-based catalysts. Detailed characterization of the spent catalysts using X-ray diffraction (XRD) and transmission electron microscopy (TEM) reveal 10 nm Co nanoparticles with hcp Co metal phase. Preliminary DFT calculations indicate the importance of the counter-ion for sodium and the consequences to catalysis. The approach of dispersing metallic Co nanoparticles on the MnO support, and utilizing alkali metal Na and its counter-ion S as electronic promoters is effective in reducing CO_2_ and methane formation, hence creating new opportunities in gas-to-chemicals processes.

## Results

### Catalysts

Co_1_Mn_3_ catalysts with an atomic ratio Co/Mn = 1/3 were synthesized via co-precipitation, and the calcined catalysts were impregnated with Na_2_CO_3_, (NH_4_)_2_SO_4_, Na_2_S_2_O_3_ or Na_2_S precursors followed by another calcination step. These catalysts were named Co_1_Mn_3_–Na_2_O, Co_1_Mn_3_,−SO_4_^2−^, Co_1_Mn_3_–Na_2_S_2_O_3_ and Co_1_Mn_3_–Na_2_S, respectively. As a comparison, Co_3_Mn_1_ catalysts were also synthesized and named in a similar fashion. An overview of calcined catalysts and their elemental loadings of Mn, Co, Na and S are included in Supplementary Table 1. The XRD pattern of calcined Co_1_Mn_3_–Na_2_S (Supplementary Figure 1) consisted of Mn_2_O_3_, MnO_2_ and CoMnO_3_ phases, and the addition of promoters did not result in change of crystalline phases. An SEM image (Supplementary Figure 2) of calcined Co_1_Mn_3_–Na_2_S, showed its morphology and the homogeneity of Co and Mn elemental loadings was confirmed by scanning electron microscopy-energy-dispersive X-ray spectroscopy (SEM-EDX, Supplementary Table 2). Scanning transmission electron microscopy-energy-dispersive X-ray spectroscopy (STEM-EDX) mapping (Supplementary Figure 3) also showed mixing of Co and Mn, and no isolated Co nanoparticles were observed.

### Catalytic performance

Catalytic performance was evaluated at a range of reaction conditions (240–280 °C, 1–10 bar, H_2_/CO = 2). At mild conditions of 240 °C, 1 bar, H_2_/CO = 2, 1% CO conversion, Co_1_Mn_3_–Na_2_S displayed a high C_2_–C_4_ olefins selectivity of 54% with a C_2_–C_4_ olefin/paraffin ratio of 17. Moreover, methane selectivity at 17% was lower than what was predicted by the ASF distribution (Supplementary Figure 4 and Supplementary Table 3). While the addition of Na_2_S improved selectivity, it also decreased activity which is in agreement with literature that S is detrimental to activity of metallic Co catalysts^30,32^. In a control experiment, addition of sulfur only (without Na) was shown to decrease activity, while increasing methane selectivity (Supplementary Figure 4 and Supplementary Table 3).

**Fig. 1:**
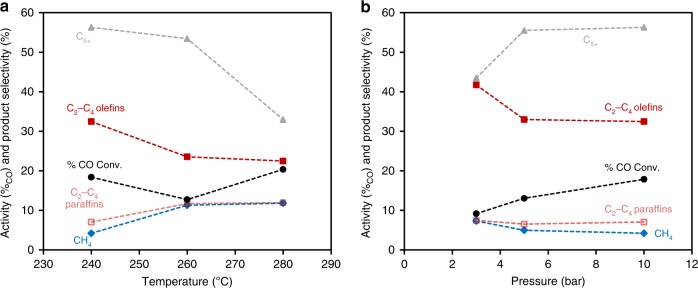
Catalytic performance of Co_1_Mn_3_–Na_2_S at different reaction temperatures or pressures. **a** Activity and selectivity at 240–280 °C, 10 bar, and H_2_/CO = 2, and **b** activity and selectivity at 240 °C, 3–10 bar, and H_2_/CO = 2. Activity is shown here as % CO conversion and product selectivity is shown in terms of methane, CH_4_ (blue solid diamonds), C_2_–C_4_ olefins (red solid squares), C_2_–C_4_ paraffins (light red open squares), and C_5+_ (grey solid triangles) which include all other products except CO_2_ and C_1_–C_4_ hydrocarbons

The effects of reaction pressures and temperatures on the catalytic performance of Co_1_Mn_3_–Na_2_S are shown in Fig. 1 and detailed information can be found in Supplementary Tables 4–7. From Fig. 1a, at 10 bar, H_2_/CO = 2, 13–30% CO conversion, an increase in temperature from 240 to 280 °C corresponded to decrease in C_2_–C_4_ olefins and C_5+_ selectivities, but increase in methane and C_2_–C_4_ paraffins selectivities. For Co_1_Mn_3_–Na_2_S 21% CO_2_ selectivity was attained at 280 °C and 10 bar. From Fig. 1b, at 240 °C, H_2_/CO = 2, 10–18% CO conversion, an increase in pressure from 3 to 10 bar corresponded to an increase in C_5+_ selectivity together with a decrease of selectivity towards C_1_–C_4_ hydrocarbon products. For Co_1_Mn_3_–Na_2_S, no CO_2_ production was detected at 3–10 bar, 240 °C, H_2_/CO = 2. The increase in chain growth probability, *α*, due to increase in pressure was confirmed by the ASF distribution plot in Supplementary Figure 5. Notably, the methane fraction was always lower than expected from the ASF distribution for Co_1_Mn_3_–Na_2_S. Since a high olefin/paraffin ratio was attained and no C_1_ olefin exists, a lowered C_1_ fraction is to be expected. Nonetheless, only with Na_2_S promotion this is actually achieved, while in literature catalysts always produce more methane than expected.

**Fig. 2:**
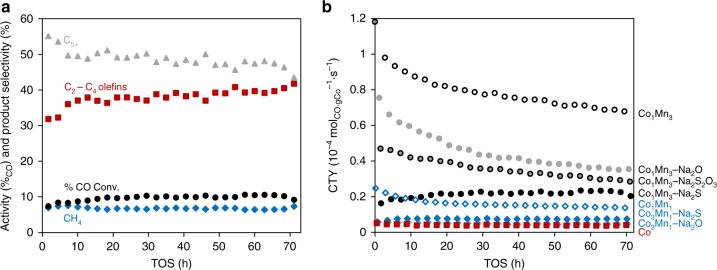
Catalytic performance over 70 h time-on-stream. Reaction conditions: 240 °C, 3 bar, and H_2_/CO = 2. **a** Activity in terms of %CO conversion (black solid circles) and selectivity towards methane, CH_4_ (blue solids diamonds), C_2_–C_4_ olefins (red solid squares), and C_5+_ (grey solid triangles) of Co_1_Mn_3_–Na_2_S over time, and **b** activity in terms of cobalt-time-yield, CTY, of various Co-based catalysts, namely Co_1_Mn_3_ (black open circles), Co_1_Mn_3_–Na_2_O (grey solid circles), Co_1_Mn_3_–Na_2_S_2_O_3_ (grey solid with black outline circles), Co_1_Mn_3_–Na_2_S (black solids circles), Co_3_Mn_1_ (blue open diamonds), Co_3_Mn_1_–Na_2_S (blue solid diamonds), Co_3_Mn_1_–Na_2_O (light blue solid diamonds), and bulk Co (red squares) over time

Catalytic stability is an important consideration hence the catalytic performance of Co_1_Mn_3_–Na_2_S over 70 h is shown in Fig. 2a. The activity of Co_1_Mn_3_–Na_2_S showed an initial increase and remained then constant over 70 h. Methane selectivity remained stable over time, while C_5+_ (all products except CO_2_ and C_1_–C_4_ hydrocarbons) and C_2_–C_4_ olefin selectivities also stabilized after 10 h. The activity and stability of Co_1_Mn_3_–Na_2_S were also compared with other Co-based catalysts in Fig. 2b. As shown in Fig. 2b, the addition of Mn increased activity for Co-based catalysts, which is in agreement with literature^34,35^. Catalysts with Co/Mn ≈0.3 showed highest activity per gram Co (cobalt-time-yield, CTY), and the addition of Na_2_O, Na_2_S_2_O_3_ or Na_2_S decreased activity. Nonetheless, the activity of Co_1_Mn_3_–Na_2_S was still higher than the remaining Co-based catalysts. In terms of stability, Co_1_Mn_3_, Co_1_Mn_3_–Na_2_O, Co_1_Mn_3_–Na_2_S_2_O_3_ and Co_3_Mn_1_ showed deactivation but all other catalysts remained stable over 70 h.

At more industrially relevant conditions of 240 °C, 10 bar, H_2_/CO = 2, 18–30% CO conversion, the catalytic performance of Co_1_Mn_3_–Na_2_S was compared to other Co-based catalysts (Table 1). Co_1_Mn_3_–Na_2_S displayed the highest selectivity towards lower olefins and lowest selectivities towards undesired methane and lower paraffins (C_2_–C_4_ olefin/paraffin ratio = 4.2). Remarkably, CO_2_ selectivity was below 3% (below detection limit, see Supplementary Figure 6 for chromatograms), suggesting the absence of WGS activity and making it an attractive catalyst for H_2_-rich syngas. CO_2_ selectivity was consistently below detection limit for all catalysts except where less Mn is present, i.e. Co_3_Mn_1_–Na_2_O and Co_3_Mn_1_–Na_2_S. Even so, the suppression of WGS activity by Na_2_S instead of Na_2_O addition was evident by the CO_2_ selectivity of Co_3_Mn_1_–Na_2_S compared to Co_3_Mn_1_–Na_2_O, i.e. 13 versus 28%, respectively. The precursor of Na/S and loading of Na were varied (Na_2_S and Na_2_S_2_O_3_) and the favourable effects on selectivity remain (Supplementary Table 1 and Table 1). Further optimization of precursor and loadings of the promoters is however outside the scope of this study.

**Table 1:** Catalytic performance at 240 °C, 10 bar, H_2_/CO = 2, 18–30% CO conversion

	CO conv., *X* (%)	CTY (10^−4^ mol_CO_. g_Co_^−1^ s^−1^)	C_1_, *S* (%)	C_2_–C_4_ olefins, *S* (%)	C_2_–C_4_ paraffins, *S* (%)	C_5+_, *S* (%)	CO_2_, *S* (%)	O/P C_2_–C_4_	*α*
Co	32	0.13	12	6	7	75	<2	0.8	0.69
Co_3_Mn_1_	31	0.14	11	17	10	62	<2	1.8	0.63
Co_3_Mn_1_–Na_2_O	20	0.09	9	14	7	42	28	1.9	0.53
Co_3_Mn_1_–Na_2_S	25	0.12	5	20	7	56	13	2.9	0.56
Co_1_Mn_3_	31	0.56	15	12	13	61	<2	0.9	0.67
Co_1_Mn_3_–Na_2_O	27	0.46	14	11	12	63	<2	0.9	0.65
Co_1_Mn_3_–Na_2_S	18	0.40	4	30	7	59	<3	4.2	0.53
Co_1_Mn_3_−Na_2_S_2_O_3_	22	0.42	7	25	12	56	<3	2.1	0.50

CO conversion (*X*, %), activity per gram of Co (CTY), product selectivity (*S*, %), The detection limit of CO_2_ selectivity is 0.5% yield, equivalent to 3% CO_2_ selectivity at 18% CO conversion.

Bulk Co catalyst had the highest *α*, therefore its main product was C_5+_ hydrocarbons. The addition of Mn–Co resulted in a lower *α*, and the addition of Na_2_O or Na_2_S further decreased *α*. Bulk Co catalyst showed typical ASF distribution deviation, whereby the C_1_ fraction is higher and the C_2_ fraction is lower than predicted. The addition of Na_2_O suppressed the C_1_ fraction, but this suppression was most prominent with the addition of Na_2_S (Supplementary Figure 5).

**Fig. 3:**
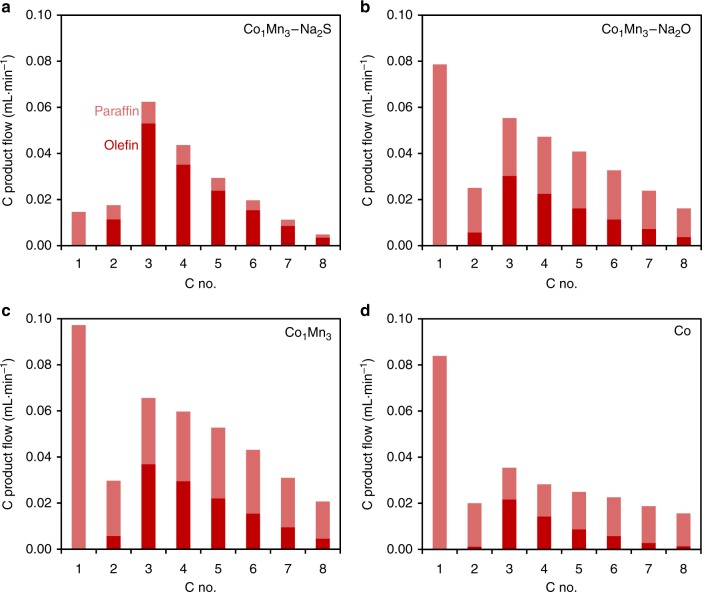
Distribution of 1-olefins and *n*-paraffins of C_1_–C_8_ hydrocarbon products. Reaction conditions: 240 °C, 10 bar, H_2_/CO = 2, 18–30% CO conversion. **a** Co_1_Mn_3_–Na_2_S, **b** Co_1_Mn_3_–Na_2_O, **c** Co_1_Mn_3_ and **d** Co. Red bar corresponds to olefin product flow and light red bar corresponds to paraffin product flow

To obtain further mechanistic insights into the various catalytic systems, the detailed C product flow of 1-olefin and *n*-paraffin for each C number product is shown in Fig. 3. The mechanistic considerations of metallic Co FT catalysts include chain growth, chain branching, primary olefin/paraffin formation and olefin secondary reactions, such as secondary hydrogenation and isomerization^36^. From Fig. 3, Co_1_Mn_3_–Na_2_S produced significantly more primary olefins than linear paraffins for each C containing hydrocarbon product. This suggests that β-H elimination was the dominant termination pathway for Co_1_Mn_3_–Na_2_S and secondary hydrogenation of olefins was also suppressed. Besides, the lower fraction of 2-butene in the C_4_ hydrocarbon product spectrum of Co_1_Mn_3_–Na_2_S implied the suppression of secondary isomerization of olefins (Supplementary Table 9 and Supplementary Figure 7). This is in agreement with the presumption that secondary hydrogenation and isomerization of olefins take place at identical sites^36^. In addition, the low methane and C_2_ hydrocarbon products from Co_1_Mn_3_–Na_2_S point to the blocking of sites for surface methyl, methylene and H species^37^.

### Structure analysis of spent catalysts

**Fig. 4:**
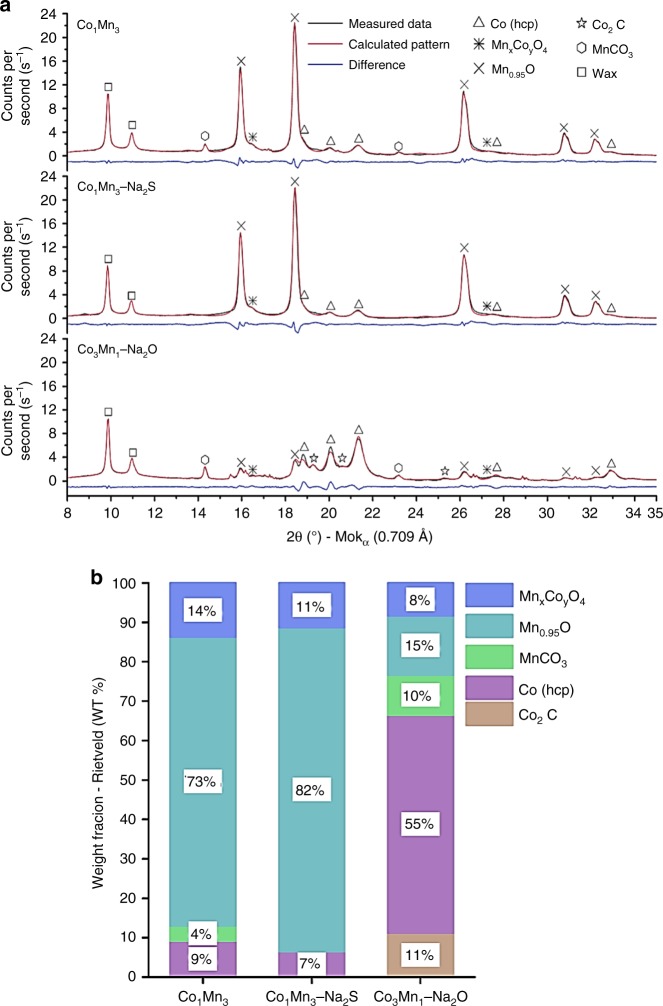
XRD analysis of spent Co_1_Mn_3_, Co_1_Mn_3_–Na_2_S, Co_3_Mn_1_–Na_2_O. Reaction conditions: 240–280 °C, 10 bar, and H_2_/CO = 2. **a** Background corrected XRD patterns and **b** rietveld QPA-based crystalline phase compositions, which shows the Mn_*x*_Co_*y*_O_4_ phase (blue), Mn_0.95_O (cyan), MnCO_3_ (green), hcp Co (violet) and Co_2_C (brown)

In order to understand the catalytic performance, the spent catalysts after being exposed to industrially relevant FTS conditions were characterized. Fig. 4a compares the XRD patterns of spent Co_1_Mn_3_, Co_1_Mn_3_–Na_2_S and Co_3_Mn_1_–Na_2_O and their crystalline phase compositions are summarized in Fig. 4b. Additional Rietveld QPA results for the spent catalysts are given in Supplementary Table 10. The diffraction patterns of crystallized wax were observed in Fig. 4a, and the wax present on the spent catalysts served to prevent oxidation of the spent catalysts. Co_1_Mn_3_ and Co_1_Mn_3_–Na_2_S consisted predominantly of a Mn_0.95_O phase, and a mixed Mn_*x*_Co_*y*_O_4_ phase was observed which both contributed most likely not to any form of FT activity. Crucially, the hexagonal (hcp) metallic Co phase was present in both spent Co_1_Mn_3_ and Co_1_Mn_3_–Na_2_S. The average crystallite size for the hcp Co phase was 9.2 nm with a standard deviation of 1.9 nm. Small contributions from a MnCO_3_ phase were also noted in both spent Co_1_Mn_3_ and Co_3_Mn_1_–Na_2_O_._ In addition to the Mn_0.95_O, Mn_*x*_Co_*y*_O_4_, Co (hcp), MnCO_3_ phases, a Co_2_C phase was present in spent Co_3_Mn_1_–Na_2_O in line with the work of Sun et al^8^.

**Fig. 5:**
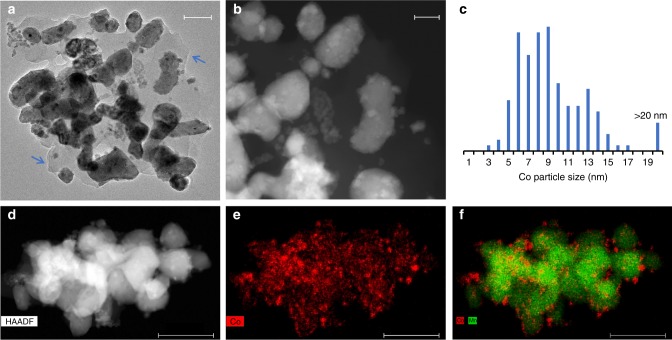
Electron microscopy images of spent Co_1_Mn_3_–Na_2_S. Reaction conditions: 240–280 °C, 10 bar, and H_2_/CO = 2. **a** bright-field TEM image with a scale bar corresponding to 100 nm, and blue arrows to point out the presence of wax, **b** dark-field TEM images with a scale bar corresponding to 50 nm, **c** particle size distribution of Co nanoparticles supported on MnO, and **d**–**f** STEM-EDX maps of Co and Mn, and the scale bars correspond to 200 nm

Fig. 5 shows the electron microscopy images and particle size distribution of spent Co_1_Mn_3_–Na_2_S after industrially relevant conditions (240–280 °C, 10 bar, and H_2_/CO = 2), and STEM-EDX mappings were carried out to differentiate Co and Mn. From Fig. 5a, wax/amorphous carbon (indicated with arrows) was observed, which is in agreement with the XRD analysis in Fig. 4a. The Co particle size distribution from TEM revealed the average Co particle size to be 9.6 nm with a standard deviation of 4.4 nm, in agreement with the Co crystallite size of 9.2 nm with a standard deviation of 1.9 nm from XRD analysis. The elemental maps of Co and Mn in Fig. 5f confirmed that spent Co_1_Mn_3_–Na_2_S consisted of Co nanoparticles well dispersed on the MnO support.

### Theoretical calculations on Na_2_S vs. Na_2_O

**Fig. 6:**
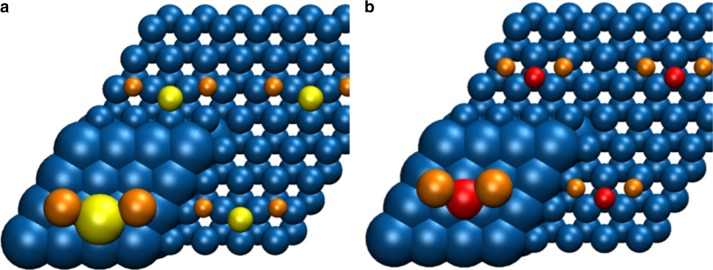
DFT-calculated binding geometries of Na_2_S and Na_2_O on the Co (0001) surface. **a** Na_2_O and **b** Na_2_S bind in a very similar fashion, although the O atom ends up above a subsurface cobalt atom and the S atom above an empty site. Atoms outside the calculation unit cell are depicted as smaller spheres; blue is Co, orange is Na, yellow is S and red is O

To gain further understanding of the difference in Na_2_S and Na_2_O, DFT calculations of both species on metallic Co (0001) surface were performed. Please note that these calculations are of a preliminary nature and further work is needed to arrive at full reaction pathway analysis which is outside the scope of this work. Pederson et al. recently performed DFT calculations on CoMnO systems for the production of light olefins and they found that selectivities can be attributed to an inhibited hydrogenation activity demonstrated by the increased barriers for CH_3_ and CH_4_ formation^22^. Strømsheim et al. recently showed that the restructuring of Co surface under CO exposure with K pre-adsorbed proceeded on the terraces rather than from the step edges^38^. Other notable theoretical studies on multiple elemental surfaces include ZnO/Cu, Co_2_C/Co, Cu/Co^39–41^. While these studies are highly relevant, they are insufficient to explain current findings. As it is shown that the combination of Na_2_S is critical for product selectivity, the theoretical calculations were focused on Na_2_S and Na_2_O promotion. The function of the sodium promoter, as any alkali metal promoter, is to donate charge to the cobalt metal. For the manganese-containing catalysts studied here, this turns out to increase olefin formation. However, for good effect another counter-ion is needed, i.e., sulfur. As shown by DFT calculations (Fig. 6), the function of the sulfur promoter is to increase the charge donation from the Na promoter ions to the cobalt surface. When no specific counter-ions are added, sodium binds in the form of Na_2_O and a considerable part of the sodium charge donation is taken up by the oxygen atom. With sulfur it is suggested to form Na_2_S instead, resulting in a higher charge donation to the cobalt surface. The DFT calculations show that every Na_2_O moiety donates a total charge of −0.51 to the cobalt surface (Na becomes +0.39 and O becomes −0.28), whereas Na_2_S donates a total charge of −0.62 (Na becomes +0.36 and S becomes −0.10). We tentatively interpret these results of higher charge donation to coincide with lower hydrogen coverages thus leading to lower methane selectivity in FTS similar to what we have reported for iron carbide^29^.

### Structure-performance relations of metallic Co vs. Co_2_C

**Fig. 7:**
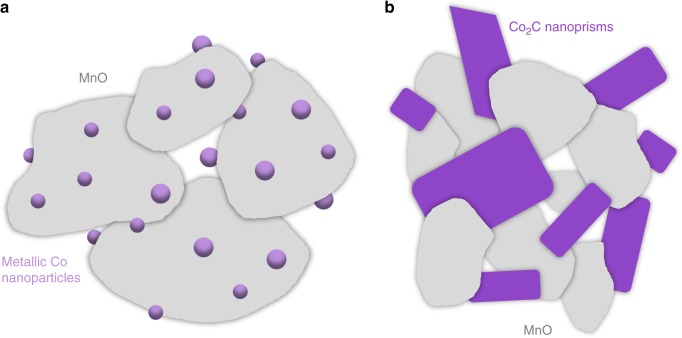
Schematic drawing of the structure of the active catalysts based on TEM and XRD analysis. **a** Co_1_Mn_3_–Na_2_S consists of metallic Co nanoparticles of ~10 nm dispersed on MnO support and **b** Co_3_Mn_1_–Na_2_O consists of Co_2_C nanoprisms of ~10–50 nm from Zhong et al^8^. Whereas the former catalyst restricts WGS and thus CO_2_ formation, the latter leads to large amounts of CO_2_

In Table 1, CO_2_ selectivity was negligible for most catalysts except Co_3_Mn_1_–Na_2_O and Co_3_Mn_1_–Na_2_S. From detailed XRD structural analysis of the spent catalysts, it was revealed that the active Co phase in Co_1_Mn_3_ and Co_1_Mn_3_–Na_2_S was metallic Co, but Co_2_C was present as an active phase in Co_3_Mn_1_–Na_2_O. For classic Co-based FT catalysts (i.e. bulk Co and CoMn) with appropriate reduction/ activation procedure and reaction conditions, metallic Co is widely accepted to be the active phase^16,42^. As metallic Co catalysts are not active for WGS, it was expected that no CO_2_ selectivity was observed for these catalysts. Upon the addition of Na_2_O or Na_2_S, the ratio of Co/Mn apparently played a critical role in influencing the crystal structure of the Co phase during FT as CO_2_ selectivities were significantly higher for Co_3_Mn_1_ than Co_1_Mn_3_. Li et al. recently concluded that Mn has a controlling effect on Co_2_C morphology and the formation of Co_2_C nanoprisms or nanospheres was dependent on the synthesis method^43^. In this study, the results of Co_3_Mn_1_–Na_2_O and Co_3_Mn_1_–Na_2_S were in agreement with Li et al. as Co content was higher than Mn in both cases. However, for catalysts with more Mn than Co, Co_2_C was not formed and Co remained in metallic phase. It is believed that when Co/Mn ≈ 0.3, MnO_*x*_ served as a support for the metallic Co nanoparticles thereby ensuring a good dispersion and stabilization of these nanoparticles (Fig. 5). MnO is also known to act as an electronic and structural promoter and the promoting effects of MnO are strongly dependent on its location and amount. For instance, Morales et al. showed that CO preferentially bonded linearly to surface metal sites when MnO loading was increased^44^. It is noted, however, that in mentioned literature, the MnO_*x*_ loading was much lower than the Co loading and the promoting effects of using MnO as a support are not yet clear. These findings are illustrated in Fig. 7.

While similar hydrocarbon product selectivities were reported earlier by Sun et al. for Na-promoted Co_2_Mn_1_ catalytic systems^8,23^, it is noteworthy to point out that in their work CO_2_ selectivity was almost 50% of CO converted due to high WGS activity. The addition of Na (most likely in Na_2_O state) served as a structural promoter and appeared to facilitate the formation of Co_2_C nanoprisms, which displayed high C_2_–C_4_ olefins and CO_2_ selectivities. In our Co_1_Mn_3_–Na_2_S catalytic system, the active phase appeared to be metallic Co and Na_2_S seemed to be an electronic promoter for product selectivity. Sun et al. showed the effect of Na_2_O loading on Co_2_Mn_1_ catalytic systems, and here we presented the importance of the counter-ion for Na (Fig. 6) using theoretical DFT calculations.

Besides the loadings and counter-ions for Na, the activation procedure is an important parameter for catalytic performance and structure-performance relations. For instance, de Smit et al. demonstrated that different Fe-carbide phases may be synthesized during catalyst pretreatment by controlling carbon chemical potential^45^. Claeys et al. concluded that while cobalt carbide is relatively stable at typical reaction conditions, it would decompose rapidly into hcp Co with hydrogen at 150 °C^46^. Davis et al. also showed that reaction conditions played a significant role in formation of cobalt carbide or metallic cobalt^47^. To induce the formation of metallic Co, calcined catalysts in our study were reduced at 350 °C and 1 bar under diluted H_2_ flow for 8 h, followed by introduction of syngas at a temperature of 180 °C and a pressure of 10 bar. This activation procedure to form metallic Co is different from that of Sun et al. to form Co_2_C^8^. To show the effect of activation procedure on catalytic performance, the same catalysts were reduced at 300 °C and 1 bar under diluted H_2_ flow for 5 h, followed by introduction of syngas at 250 °C and 10 bar. With this activation procedure, CO_2_ selectivity increased to 6% (Supplementary Table 13) possibly related to cobalt carbide formation. Nonetheless, Na_2_S was still the most effective promoter (Supplementary Tables 11–13).

In summary, we have designed a catalytic system Co_1_Mn_3_–Na_2_S, which showed negligible WGS activity and suppression of methane formation in FTS. Structure analysis of the spent catalyst revealed 10 nm metallic Co nanoparticles as the active phase supported on MnO during reaction. Theoretical calculations revealed the importance of counter-ion S for Na, and Na_2_S was more efficient in tuning hydrocarbon product selectivity than Na_2_O. Tentatively the addition of Na_2_S to Co_1_Mn_3_ was proposed to deactivate sites for secondary olefin hydrogenation and isomerization and for methanation, whereas the lower degree of alkalization as compared with Na_2_O is insufficient to promote the WGS reaction. For this complex catalytic system, further studies on the effects of various elements on structure-performance relations and advanced characterization are advocated.

The state-of-the-art processes and catalysts for direct production of lower olefins from synthesis gas are compared in Supplementary Table 14. While all catalysts showed favourable selectivity towards lower olefins, Co_1_Mn_3_–Na_2_S is the only catalyst, which combined lower olefin selectivity with negligible CO_2_ production. This comparison suggests that Co_1_Mn_3_–Na_2_S is a promising catalyst which is capable of producing chemicals and fuels directly from H_2_-rich syngas derived from natural gas. This gas-to-chemicals process would greatly reduce CO_2_ emissions, thereby contributing prevention of climate change.

## Methods

### Synthesis of CoMn catalysts

Two grams of Co(NO_3_)_2_•6H_2_O (99 +%, Acros) and 5.7 g Mn(NO_3_)_2_•4H_2_O (97.5 +%, Acros), were dissolved in 40 mL deionized water at room temperature in a 100 mL round-bottom flask. After 1 h of stirring at room temperature, the round-bottom flask was heated to 60 °C in a water bath. Twenty microliters of 1.0 M aqueous (NH_4_)_2_CO_3_ (30 +% (NH_3_), Acros) was added dropwise to the mixed nitrate solution using a mechanical pump set at 1 mL/min and pH was kept at ~8. The resulting pink powder was aged for 30 min at room temperature, followed by decanting and washing with deionized water thrice. The precipitate was then dried at 120 °C under static air for 2 h with stirring every 0.5 h and calcined at 400 °C (2 °C/min) under air flow for 2 h. The synthesized Co_1_Mn_3_ was then impregnated with either Na_2_CO_3_ anhydrous (99.5%, Fisher Scientific), (NH_4_)_2_SO_4_ (≥99.0%, Sigma-Aldrich), Na_2_S_2_O_3_ anhydrous (≥98.0%, Sigma-Aldrich) or Na_2_S nonahydrate (≥98.0%, Sigma-Aldrich) precursor's dissolved in deionized water, followed by calcination at 400 °C (2 °C/min) under air flow for 2 h. The Co_3_Mn_1_ catalysts were synthesized with the identical procedure but different Co and Mn precursors mass loadings.

### Catalyst characterization

Elemental loading of Co, Mn, Na and S were determined with a Thermo Jarrell Ash model ICAP 61E trace analyzer inductively coupled plasma-atomic emission spectrometer (ICP-AES). Scanning electron microscopy (SEM) images were taken using a FEI XL30 FEG SEM instrument in backscattering electron mode at an acceleration voltage of 15 kV. SEM samples were prepared on carbon grids followed by Pt-coating to improve electron conductivity. STEM-HAADF images and EDX analysis were obtained with an FEI Talos F200X transmission electron microscope, operated at 200 kV and equipped with a high-brightness field emission gun (X-FEG) and a Super-X G2 EDX detector. More than 150 particles were measured to obtain a particle size distribution. XRD patterns were measured with a Bruker D8 Discover instrument in Debye-Scherrer transmission (capillary) geometry with a Mo (*K*_*α*1_ 0.709 Å) source. A Göbel-mirror was used to focus a near-parallel X-ray beam on the 1000 µm (OD, wall thickness 10 µm) capillary. Energy dispersive LynxEye XE Position Sensitive Detector (PSD) was used, only accepting diffracted X-ray photons originating from Mo *K*_*ɑ*_ emission lines. Details on the instrument can be found in a recent publication^48^. Measurement parameters used were 2θ 5–48° with step size of 0.032° and exposure time of 18 s per step, for each measurement. Rietveld Quantitative Phase Analysis (Rietveld QPA) was performed on the measured diffractograms using Bruker TOPAS (v5) software. Details and discussion on the Rietveld refinement procedure are given in Supplementary Methods. Phase identification from diffractograms was done using ICDD PDF-4+2016 database and structures used in the Rietveld QPA were obtained from the same database and are listed in Supplementary Table 10.

### Catalytic tests at mild conditions

Low pressure tests were carried out at 240 °C, 1 bar, H_2_/CO = 2 v/v, <3% CO conversion. A fixed-bed reactor was loaded with 0.02 g (75–150 μm) catalyst and 0.20 g SiC (212–425 μm) for bed dilution. The catalysts were reduced prior to reaction at 350 °C (5 °C /min) under diluted H_2_ flow (33 vol.% H_2_, 67 vol.% He, 60 mL/min total flow) for 2 h. After reduction, temperature was decreased to 240 °C (2 °C /min) under 40 mL/min He flow. At 240 °C and 1 bar, the feed flow was switched to a mixture of H_2_ and CO (H_2_/CO = 2 v/v, 9 mL/min total flow). Hydrocarbons (C_1_–C_16_) from the product stream were analysed online with gas chromatography (Varian CP3800), and CO_2_ was not measured. The line from the reactor to GC was heated to at least 150 °C to prevent hydrocarbon condensation. Activities and product selectivities were calculated on a carbon atom basis. Activity is reported as moles of CO converted per gram Co per second, and moles of CO converted is based on moles of C in the hydrocarbon product stream. Product selectivity was calculated as equivalent of carbon atoms in a product with respect to the total carbon atoms present in the hydrocarbons produced (% C).

### Catalytic tests at industrially relevant conditions

Medium pressure tests were performed using a high throughput 16 parallel fixed-bed reactors set-up (Flowrence, Avantium). Each reactor was loaded with 50 mg catalyst (75–150 μm) and 100 μL SiC (212–425 μm) as diluent. The catalysts were first dried at 100 °C (5 °C /min) under He flow for 2 h and subsequently reduced at 350 °C (1 °C/min) under dilute H_2_ flow (25 vol.% H_2_, 75 vol.% He) for 8 h. After reduction, temperature was decreased to 180 °C (1 °C /min) and pressure was increased to 10 bar under H_2_ flow. At 180 °C and 10 bar, the feed flow was switched to syngas mixture (H_2_/CO/He = 60/30/10, 6.6 mL/min total flow per reactor) and subsequently the temperature was raised to 240 °C (1 °C /min). The product stream was analysed using online gas chromatography (Agilent 7890A) with Ar as carrier gas. Hydrocarbons (C_1_–C_9_) were separated on an Agilent J&W PoraBOND Q column, detected using an FID detector and quantified against the TCD signal of the internal standard He. The permanent gases (CO, H_2_, He, CO_2_ and CH_4_) were separated on a ShinCarbon ST (#19043) column and quantified against He as an internal standard using a TCD detector. CO_2_ was also measured and the detection limit of CO_2_ was determined to 0.5% yield, which was 3% CO_2_ selectivity and 18% CO conversion (Supplementary Figure 5 and Supplementary Table 8). Catalytic activity and product selectivities were measured at 240–280 °C, 10 bar, H_2_/CO = 2, 10–70% CO conversion. To show the effect of activation procedure on catalytic performance, the same catalysts were reduced at 300 °C and 1 bar under diluted H_2_ flow for 5 h, followed by introduction of syngas at 250 °C and 10 bar. Definitions of the selectivity and activity, expressed as CO conversion and cobalt-time-yield (CTY) are included as Supplementary Methods.

### DFT calculations

DFT modelling was performed with the ADF-BAND package (version 2016.102)^49,50^, using the rPBE functional^51^ and Grimme D3 corrections^52^. A TZP basis set with small frozen cores, a “good” k-space, and otherwise “normal” settings were used. For efficiency, the SCF was converged to only 5 × 10^−4^ Hartree. Gradients were converged to 0.001 Hartree/Å. The bulk cobalt unit cell vectors were reoptimized, giving *a* = 2.43 Å (experimental 2.51 Å) and *c* = 3.91 Å (experimental 4.07 Å). The (0001) surface was modelled with 6 atomic layers, giving a slab of 12 Å thick, of which the bottom two layers were frozen and calculated at minimal settings (SZ basis set with large frozen core, orbital confinement to 4 bohr, and “basic” settings for the Becke grid and zlm-fit parameters). The surface unit cell consisted of 4 × 4 atoms. Since ADF-BAND uses true 2D periodicity, no vacuum spacing nor dipole corrections were needed. Atomic charges were calculated with Hirshfeld’s method^53^.

## Supplementary information

Supplementary Information

Peer Review File

## Data Availability

The datasets generated during and/or analysed during the current study are available from the corresponding author on reasonable request.
