# Supplementary material for: Promoted cobalt metal catalysts suitable for the production of lower olefins from natural gas
Source: Nat Commun. 2019 Jan 11;10:167. doi: 10.1038/s41467-018-08019-7 (PMC6329823; doi:10.1038/s41467-018-08019-7)
Supplement: Supplementary file 1 — Supplementary Information [file 41467_2018_8019_MOESM1_ESM.pdf]

Supplementary Information

Promoted Cobalt Metal Catalysts Suitable for the Production of Lower Olefins from Natural Gas

Xie *et al.*

Contents

Supplementary Figures 2
Supplementary Tables 9
Supplementary Methods 23
Supplementary References 25

Supplementary Figures

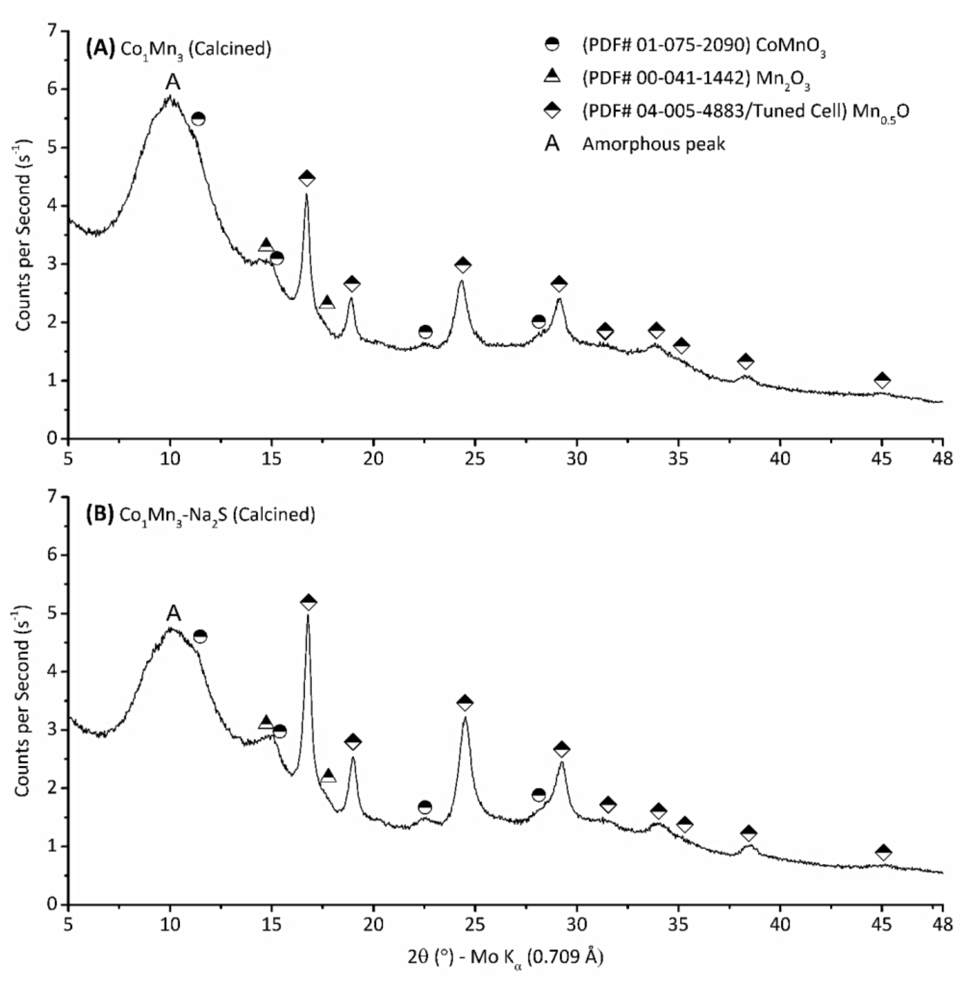

Supplementary Figure 1. XRD patterns of calcined catalysts. a, Co_1Mn_3 and **b,** Co_1Mn_3-Na_2S XRD patterns showed no significant differences after calcination at 400 °C under air flow for 2 h.

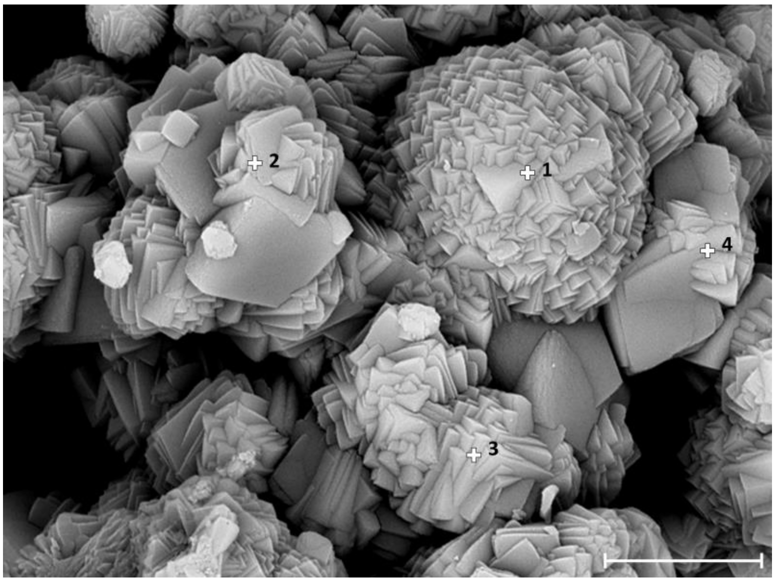

Supplementary Figure 2. SEM image of Co_1Mn_3-Na_2S and locations where EDX measurements were made. The scale bar corresponds to 3 μm. Relative elemental loadings of Co_1Mn_3 determined by SEM-EDX were recorded in Supplementary Table 2.

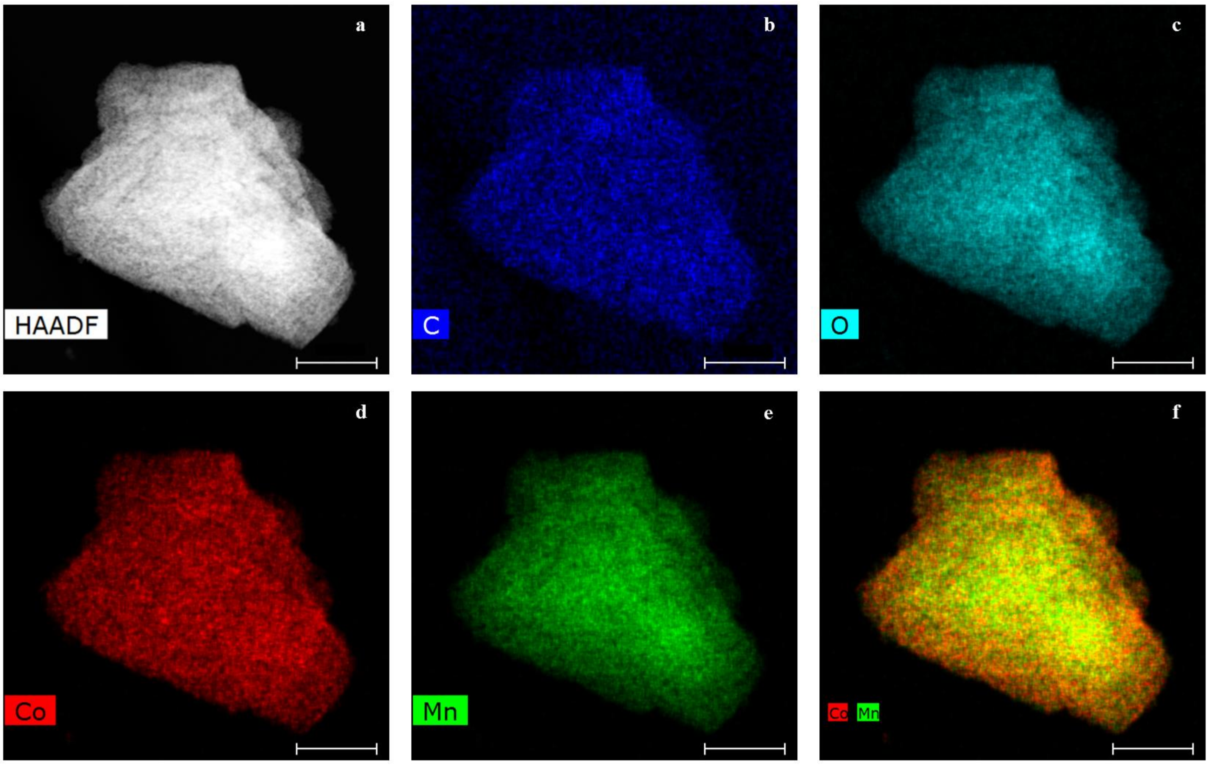

Supplementary Figure 3. STEM-HAADF image and STEM-EDX mappings of calcined Co_1Mn_3-Na_2S. a, STEM-HAADF image and **b, c, d, e, f,** elemental mapping of C, O, Co, Mn and Co/Mn respectively. The scale bar corresponds to 200 nm.

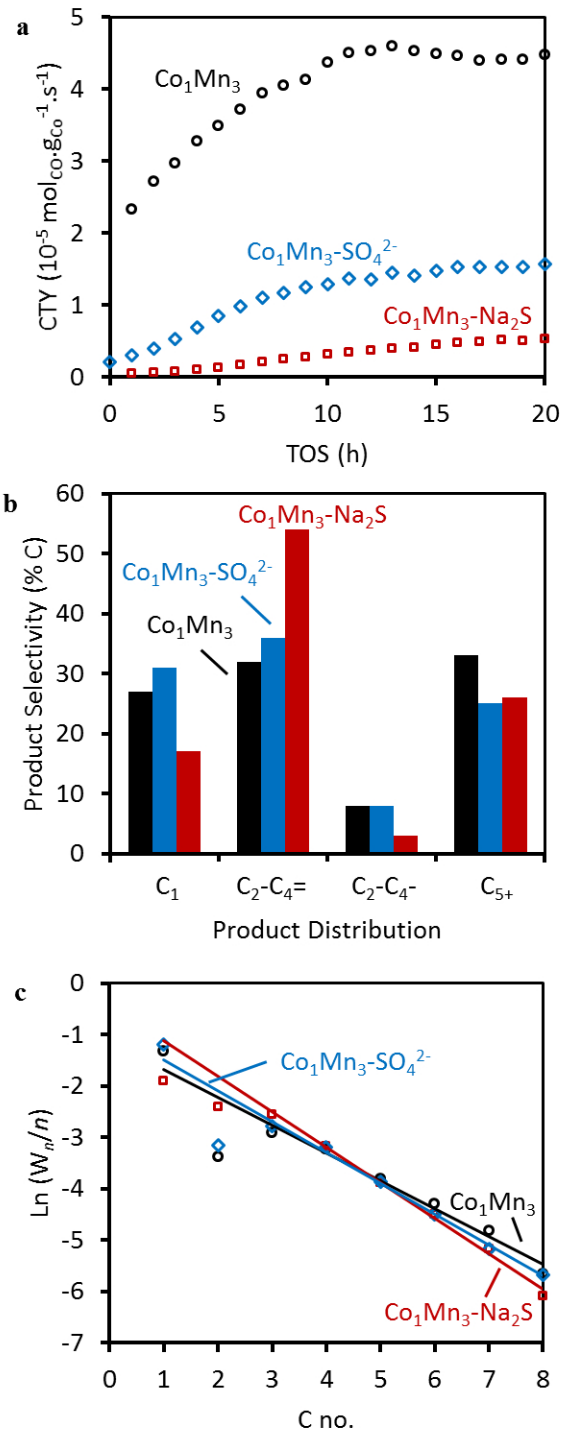

Supplementary Figure 4. Catalytic performance at 240 °C, 1 bar, H_2/CO = 2, < 10 % CO conversion. a, activity over time of Co_1Mn_3 (black open circles), Co_1Mn_3-SO_4^{2-} (blue open diamonds) and Co_1Mn_3-Na_2S (red open squares), **b,** product selectivity after 20 h and **c,** ASF distributions after 20 h. α is calculated using the gradient of C_3 to C_8.

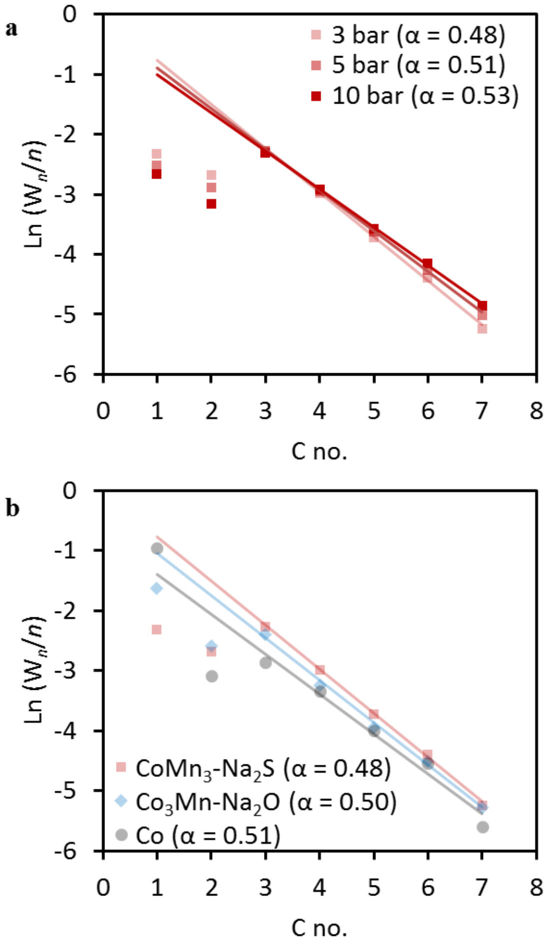

Supplementary Figure 5. ASF distributions at 240 °C, H_2/CO = 2. a, $CoMn_3$-Na_2S at different pressures (3 – 10 bar, higher pressure corresponds to darker red squares) and **b,** Co (black circles), Co_1Mn_3-Na_2O (blue diamonds) and Co_1Mn_3-Na_2S (red squares) at 3 bar and 10 % CO conversion. *α* is calculated using the gradient of C_3 to C_7.

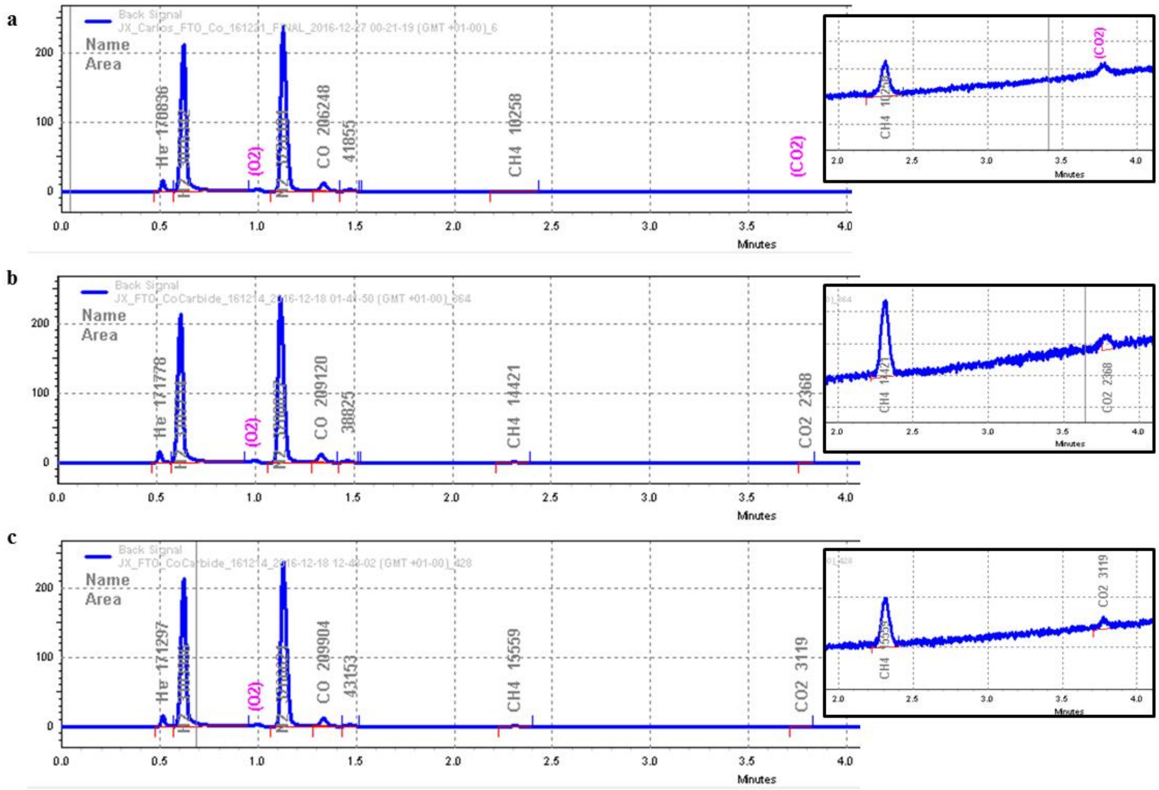

Supplementary Figure 6. TCD chromatograms at different CO conversions to show CO_2 sensitivity. a, at 18 % CO conversion (CO_2 yield is below the detection limit), obtained with Co_1Mn_3-Na_2S with 'metallic Co' activation procedure, 240 °C, 10 bar, H_2/CO = 2. **b,** at 17 % CO conversion with 3 % CO_2 selectivity, obtained with Co_1Mn_3-Na_2S with 'Co Carbide' activation procedure, 240 °C, 3 bar, H_2/CO = 2. **c,** at 17 % CO conversion with 5 % CO_2 selectivity, obtained with Co_1Mn_3-Na_2S with 'Co Carbide' activation procedure, 240 °C, 3 bar, H_2/CO = 2. A summary of the peak areas can be found in Supplementary Table 8.

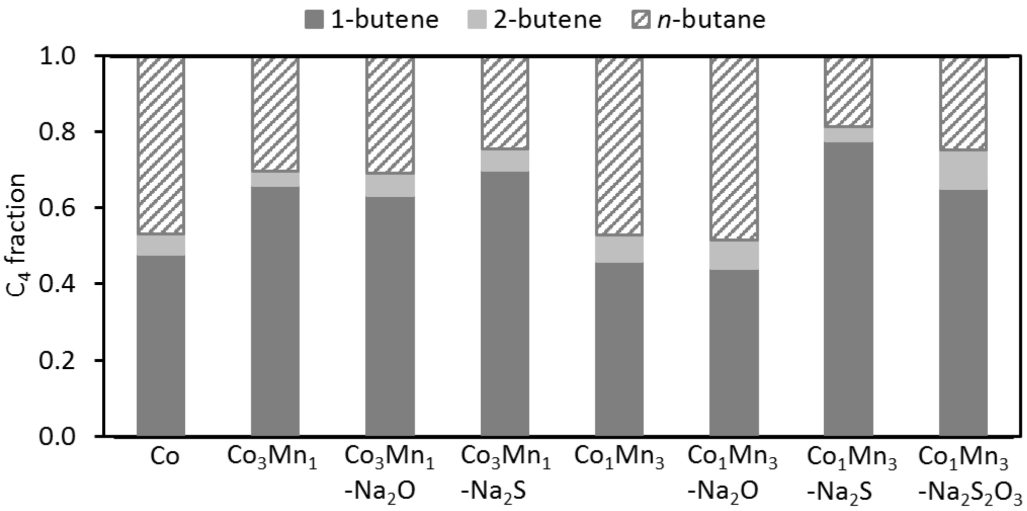

Supplementary Figure 7. Fraction of C_4 products produced by different catalysts at 240 °C, 10 bar, H_2/CO = 2, 18 – 30 % CO conversion. Fraction of C_4 products which was 1-butene (dark grey, solid), 2-butene (light grey, solid) and *n*-butane (dark grey, strips)

Supplementary Tables

Supplementary Table 1. List of catalysts with their elemental loadings and atomic ratios measured with ICP-AES.

	% Weight				Atomic Ratio			
	Co	Mn	Na	S	Mn/Co	S/Co	Na/Co	Na/S
Co	77.75	0.00	0.01	0.00				
Mn	0.00	68.77	0.02	0.12				
Co_3Mn_1	58.38	16.23	0.03	0.03	0.30	0.00	0.00	1.06
Co_3Mn_1-Na_2O	57.91	16.13	0.25	0.03	0.30	0.00	0.01	10.40
Co_3Mn_1-Na_2S	57.27	16.04	0.24	0.21	0.30	0.01	0.01	1.61
Co_1Mn_3	15.03	52.38	0.02	0.09	3.74	0.01	0.00	0.23
Co_1Mn_3-Na_2O	15.55	54.21	0.07	0.09	3.74	0.01	0.01	1.04
Co_1Mn_3-Na_2S	15.81	54.91	0.08	0.14	3.73	0.02	0.01	0.78
Co_1Mn_3-$Na_2S_2O_3$	15.54	52.59	0.04	0.13	3.63	0.02	0.01	0.45

Supplementary Table 2. Relative elemental loadings of Co_1Mn_3 determined by SEM-EDX, the specific locations measured were indicated in Supplementary Figure 2.

	Intensity Counts						Atomic ratio Mn/Co
	C (K)	Pt (M)	Pt (L)	O (K)	Mn (K)	Co (K)	
1	30.57	84.83	9.83	336.30	180.90	39.67	3.13
2	33.17	78.17	9.13	330.53	176.70	43.90	2.75
3	38.20	88.93	7.97	371.03	178.83	41.43	2.94
4	27.73	65.27	4.60	356.20	191.70	40.20	3.20

C is due to the carbon grid used to prepare the sample, Pt was added to the sample to improve the electron conductivity of the sample.

Supplementary Table 3. Catalytic Performance of Co_1Mn_3 and Co_1Mn_3-Na_2S at 240 °C, 1 bar, H_2/CO = 2, < 10 % CO conversion, time = 20 h.

	CO conv. (%)	CTY (10^{-5} $mol_{CO}.g_{Co}^{-1}.s^{-1}$)	O/P (C_2-C_4)	α	Product Selectivity (% C)			
					CH_4	C_2– C_4 =	C_2–C_4 -	C_{5+}
Co_1Mn_3	6.5	3.3	4.1	0.58	27	32	8	33
Co_1Mn_3-SO_4^{2-}	2.3	1.6	4.7	0.55	31	36	8	25
Co_1Mn_3-Na_2S	0.8	0.4	16.5	0.52	17	54	3	26

Supplementary Table 4. Catalytic Performance of Co_1Mn_3-Na_2S at 240 - 280 °C, 10 bar, H_2/CO = 2, TOS = 20 h.

	CO conv. (%)	CTY (10^{-4} $mol_{CO}/g_{Co}^{-1}.s^{-1}$)	Selectivity (%C)					O/P C_2-C_4
			C_1	C_2-C_4 =	C_2-C_4 -	C_{5+}	CO_2	
240	16	0.3	4	32	7	56	< 3	4.6
260	13	0.3	11	24	12	53	< 4	2.0
280	20	0.4	12	22	12	33	21	2.0

Supplementary Table 5. Catalytic Performance of Co_1Mn_3 at 240 - 280 °C, 10 bar, H_2/CO = 2, TOS = 20 h.

	CO conv. (%)	CTY (10^{-4} $mol_{CO}/g_{Co}^{-1}.s^{-1}$)	Selectivity (%C)					O/P
			C_1	C_2-C_4 =	C_2-C_4 -	C_{5+}	CO_2	C_2-C_4
240	26	0.5	7	25	9	59	< 2	2.6
260	66	1.3	23	3	17	51	6	0.2
280	75	1.5	29	4	17	41	9	0.3

Supplementary Table 6. Catalytic performance at 240 °C, 3 bar, H_2/CO = 2.

	CO conv. (%)	CTY (10^{-4} mol_{CO}. $g_{Co}^{-1}.s^{-1}$)	Product Selectivity (%C)					O/P C_2-C_4	α
			C_1	C_2-C_4 =	C_2-C_4 -	C_{5+}	CO_2		
Co	10	0.04	29	11	19	41	<5	0.6	0.51
Co_3Mn_1	29	0.14	16	15	8	61	<2	1.9	0.68
Co_3Mn_1-Na_2O	10	0.05	6	15	4	42	33	3.9	0.50
Co_3Mn_1-Na_2S	15	0.08	6	26	6	47	14	4.1	0.52
Co_1Mn_3	37	0.68	11	14	7	68	<2	1.9	0.71
Co_1Mn_3-Na_2O	21	0.36	9	18	6	67	<3	2.8	0.66
Co_1Mn_3-Na_2S	10	0.20	7	42	7	43	<5	5.6	0.48
Co_1Mn_3-$Na_2S_2O_3$	15	0.28	9	28	9	54	<3	3.1	0.56

Supplementary Table 7. Catalytic performance at 240 °C, 5 bar, H_2/CO = 2.

	CO conv. (%)	CTY (10^{-4} mol_{CO}. g_{Co}^{-1}.s^{-1})	Product Selectivity (%C)					O/P C_2-C_4	α
			C_1	C_2-C_4 =	C_2-C_4 -	C_{5+}	CO_2		
Co	15	0.06	22	10	16	52	<3	0.6	0.65
Co_3Mn_1	31	0.14	12	15	7	65	<2	2.1	0.68
Co_3Mn_1-Na_2O	13	0.06	8	15	5	42	30	2.8	0.5
Co_3Mn_1-Na_2S	19	0.09	5	22	6	50	16	3.7	0.54
Co_1Mn_3	33	0.61	11	14	9	66	<2	1.5	0.69
Co_1Mn_3-Na_2O	22	0.38	16	13	10	62	<3	1.3	0.66
Co_1Mn_3-Na_2S	13	0.29	5	33	7	56	<4	5.1	0.51
Co_1Mn_3-$Na_2S_2O_3$	18	0.35	7	29	9	55	<3	3.2	0.52

Supplementary Table 8. Summary of TCD peak areas from TCD Chromatograms, Supplementary Figure 6.

	TCD Peak Area				% CO Conversion	% CO_2 Selectivity	% CO_2 Yield
	He	CO	CH_4	CO_2			
a	170836	206248	10258	-	18		
b	171778	209120	14421	2368	17	3	0.5
c	171297	209904	15559	3119	17	5	0.9

Supplementary Table 9. Fraction of C_4 products produced by different catalysts at varied reaction conditions. Extension of Table 1, Supplementary Table 4, 5, 6 and 7.

	Pressure (bar)	Temp. (°C)	Fraction of C_4 products		
			n-butane	1-butene	2-butene
Co	3	240	0.63	0.29	0.09
	5	240	0.64	0.29	0.07
	10	240	0.47	0.48	0.05
Co_3Mn_1	3	240	0.26	0.66	0.08
	5	240	0.26	0.69	0.04
	10	240	0.30	0.66	0.04
Co_3Mn_1-Na_2O	3	240	0.13	0.81	0.06
	5	240	0.18	0.75	0.07
	10	240	0.31	0.63	0.06
Co_3Mn_1-Na_2S	3	240	0.14	0.76	0.11
	5	240	0.16	0.75	0.09
	10	240	0.25	0.70	0.06
Co_1Mn_3	3	240	0.27	0.60	0.13
	5	240	0.33	0.58	0.10
	10	240	0.47	0.46	0.07
	10	260	0.82	0.10	0.08
	10	280	0.73	0.13	0.14
Co_1Mn_3-Na_2O	3	240	0.19	0.72	0.09
	5	240	0.36	0.53	0.11
	10	240	0.48	0.44	0.08
Co_1Mn_3-Na_2S	3	240	0.11	0.81	0.07
	5	240	0.16	0.79	0.05
	10	240	0.19	0.78	0.04
	10	260	0.32	0.61	0.07
	10	280	0.23	0.55	0.22
Co_1Mn_3-$Na_2S_2O_3$	3	240	0.16	0.70	0.14
	5	240	0.16	0.73	0.10
	10	240	0.26	0.65	0.09

Supplementary Table 10. Results for the Rietveld Quantitative Phase Analysis from the measured X-ray powder diffraction patterns on the spent catalysts.

	R_{wp},[1)]	% Weight	L_{vol}[2)] (nm)	Max local strain ε_0 (10^{-4})	PDF 2016 #[3)]
Co_1Mn_3	8.86				
$Mn_XCo_yO_4$		13.7(7)	4.5(3)	-	04-009-3195[4)]
$Mn_{0.95}O$		73.3(7)	27.2(6)	11.1(3)	04-002-8161
$MnCO_3$		3.8(3)	48(18)	6(4)	00-044-1472
Co (hcp)		9.2(4)	6.6(6)	20(5)	04-001-3273
Co_1Mn_3-Na_2S	11.71				
$Mn_XCo_yO_4$		11.3(7)	5.7(5)	-	04-009-3195[4)]
$Mn_{0.95}O$		82.2(7)	23.6(6)	10.6(4)	04-002-8161
Co (hcp)		6.5(4)	9.2(19)	32(6)	04-001-3273
Co_3Mn_1-Na_2O	9.26				
$Mn_XCo_yO_4$		8.3(12)	3.2(7)	-	04-009-3195[4)]
$Mn_{0.95}O$		15.1(17)	17.9(17)	11(2)	04-002-8161
$MnCO_3$		10.1(4)	41(9)	0(11)	00-044-1472
Co (hcp)		55.3(10)	6.36(17)	0(3)	04-001-3273
Co_2C		11.2(5)	10.5(14)	0(3)	04-004-4639

Errors are as Standard (Absolute) Uncertainty. In used short-hand notation for error e.g. 15.1(17) means 15.1±1.7 and 48(18) is 48±18

1) Background subtracted weighted profile R-factor.

2) Volume weighted mean column length (i.e. volume weighted "crystallite size").

3) ICDD PDF-4+ 2016 database reference number for the phase structures used in the fitting.

4) Nominal structure is $Mn_{1.6}Co_{1.4}O_4$, see fitting details discussion (Supplementary Methods)

Supplementary Table 11. Catalytic performance at 240 °C, 3 bar, H_2/CO = 2 with 'carbide' activation procedure.

	CO conv. (%)	CTY (10^{-4} $mol_{CO}.g_{Co}^{-1}.s^{-1}$)	Product Selectivity (%C)					O/P
			C_1	C_2-C_4 =	C_2-C_4 -	C_{5+}	CO_2	C_2-C_4
Co	28	0.09	20	8	17	55	0	0.4
Co_3Mn_1	47	0.22	13	8	14	62	3	0.6
Co_3Mn_1-Na_2O	25	0.12	11	17	9	40	23	1.8
Co_3Mn_1-Na_2S	24	0.11	12	20	14	39	14	1.4
Co_1Mn_3	33	0.59	8	14	6	72	0	2.5
Co_1Mn_3-Na_2O	16	0.28	10	20	8	52	10	2.5
Co_1Mn_3-Na_2S	17	0.28	7	30	7	51	5	4.3
Co_1Mn_3-$Na_2S_2O_3$	21	0.37	10	18	6	63	3	2.8

Supplementary Table 12. Catalytic performance at 240 °C, 5 bar, H_2/CO = 2 with 'carbide' activation procedure.

	CO conv. (%)	CTY (10^{-4} $mol_{CO}.g_{Co}^{-1}.s^{-1}$)	Product Selectivity (%C)					O/P
			C_1	C_2-C_4 =	C_2-C_4 -	C_{5+}	CO_2	C_2-C_4
Co	27	0.09	21	8	17	54	0	0.5
Co_3Mn_1	37	0.18	14	10	16	58	3	0.6
Co_3Mn_1-Na_2O	34	0.16	10	16	9	44	22	1.8
Co_3Mn_1-Na_2S	30	0.14	11	19	14	42	14	1.4
Co_1Mn_3	31	0.56	16	11	10	61	2	1.1
Co_1Mn_3-Na_2O	18	0.30	14	17	10	49	10	1.6
Co_1Mn_3-Na_2S	21	0.35	6	27	8	54	5	3.6
Co_1Mn_3-$Na_2S_2O_3$	20	0.35	8	22	8	58	4	2.7

Supplementary Table 13. Catalytic performance at 240 °C, 10 bar, H_2/CO = 2 with 'carbide' activation procedure.

	CO conv. (%)	CTY (10^{-4} $mol_{CO}.g_{Co}^{-1}.s^{-1}$)	Product Selectivity (%C)					O/P
			C_1	C_2-C_4 =	C_2-C_4 -	C_{5+}	CO_2	C_2-C_4
Co	45	0.16	15	6	10	70	0	0.6
Co_3Mn_1	41	0.19	16	11	16	52	4	0.7
Co_3Mn_1-Na_2O	51	0.24	10	13	9	46	22	1.4
Co_3Mn_1-Na_2S	41	0.19	9	16	12	48	15	1.3
Co_1Mn_3	29	0.51	16	10	13	62	0	0.8
Co_1Mn_3-Na_2O	22	0.38	12	13	12	54	9	1.1
Co_1Mn_3-Na_2S	30	0.51	5	23	8	58	6	3.0
Co_1Mn_3-$Na_2S_2O_3$	30	0.52	7	19	11	59	4	1.8

Supplementary Table 14. Comparison of state-of-the-art processes and catalysts for the direct production of lower olefins from synthesis gas.

	T (°C)	P (bar)	H_2/CO	MTY (10^{-5} $mol_{CO}/g_M.s$)	CO_2 sel. (%)	Product Sel. (% C, CO_2 free)			
						CH_4	C_2– C_4 =	C_2–C_4 -	C_{5+}
Co/MnO$_x$/Na$_2$S	240	10	2	4.3	<3	4	33	7	56
Co_2C nanoprisms [1]	250	10	0.5	3.8	47	5	32	6	58
Fe/Na/S/alumina [2]	340	20	1	2.6	41	17	39	19	25
ZnCrO$_x$/MSAPO [3]	400	25	1.5	-	45	5	74	14	7

Activity per gram of metal (MTY), C_{5+} includes C_{5+} hydrocarbons and all oxygenates

Supplementary Methods

Quantitative Phase Analysis with the Rietveld Method – Fitting Details

Instrumental line broadening and emission profile were solved using National Bureau of Standards Standard Reference Material 640 (Si, a=5.43088 Å). For solving the instrumental line broadening, Fundamental Parameters approach implemented in TOPAS was used. Shifts in natural Mo K-emission lines introduced by the used GöbelMirror were corrected by fixing the $K_{\alpha1}$ emission to its natural value of 0.709300 Å and refining the $K_{\alpha2}$ line to an arbitrary value of 0.713467 Å (natural Mo $K_{\alpha2}$ 0.713607 Å) for the best peak fit on the used Si reference standard. 2 broad, low intensity K_{α} were added to the emission profile to account for the asymmetry from the multiplet K-emission lines.

Structures for fitting the diffractograms were obtained from ICDD PDF-4+ 2016 database and their reference numbers are tabulated per phase in Supplementary Table 10. Database structures for the identified phases provided a good model for the fitting, with the exception of $Mn_xCo_yO_4$ phase. Best fit for the observed Mn_xCo_y composite oxide phase was obtained with using nominal structure for $Mn_{1.6}Co_{1.4}O_4$ (PDF # 04-009-3195). However, lattice parameters deviated strongly from the values reported in the database, a=5.803 Å and c=8.683 Å versus values used for fitting a=5.783 Å and c=8.900 Å. For $Mn_xCo_yO_4$ phases, the phase structure (and observed diffraction peak positions) strongly depend on the Mn:Co ratio.[4] Thus, the exact Mn:Co ratio for the phase is unknown and notation $Mn_xCo_yO_4$ is used and $Mn_xCo_yO_4$ phase content is an estimate. However, as Mn and Co are relatively close in their electron density and chemical properties, it is likely that the refined scale factor and the structure used for the $Mn_xCo_yO_4$ phase quantification is within reasonable accuracy needed for this work. Lattice parameters for rest of the used structures refined within ≈0.03 Å per lattice direction from the database values and the structures were considered accurate.

Crystalline wax phase was identified with database number 00-050-2246 ($C_{46}H_{94}$ n-Hexatetracontane). No solved structures exist in the PDF-4+ database for crystallized wax, therefore peaks (2) identified as crystallized wax were fitted with arbitrary (mathematical) peak phase and excluded from the quantified phase fractions. In similar fashion, broad arbitrary peaks were used to fit amorphous phase (broad hump between ≈8-12 2θ Mo K_{α}). Small amounts of residual SiC (identified by abnormally sharp diffraction peaks) from sample dilution in the catalytic testing were also fitted with individual peaks when needed, and excluded from the phase quantification.

Sample displacement, cylindrical 2θ correction (Sabine), lattice parameters per structure, background (5th order Chebyshev polynomial) and diffraction peak size-strain broadening were refined simultaneously. A single Lorentzian peak was used for the size broadening and a single Gaussian peak for strain broadening. From the Lorentzian peak, size broadening was obtained using integral breadth (i.e. crystallite shape factor k=1) and from the broadening volume weighted mean column length (L_{vol}) was calculated using the Scherrer equation. Maximum local strain ε_0 was calculated from the Gaussian peak broadening as $\varepsilon_0=\beta/4\tan(\theta)$ where β is diffraction angle θ dependent Gaussian component peak (Full-Width Half-Maximum) broadening.

In case of Co (hcp) structure, also preferred orientation was refined in lattice directions (0 1 0) and (0 0 2) using the March-Dollase algorithm as the relative database peak intensities resulted in a poor fit. By manually rotating the sample capillary, no changes were observed in the diffraction intensities, thus preferred orientation is due to physical shape of the Co (hcp) crystallites not due to imperfect Debye-Scherrer rings. The crystallite shape observed for Co (hcp) could possibly be rod-, rectangular- or plate-like.

Definitions of catalytic activity and selectivity

The activity of the catalysts is expressed as CO conversion and cobalt time yield (CTY). The CO conversion is defined according to (Supplementary Equation 1) and CTY according to (Supplementary Equation 2).

$$X_{\mathrm{CO}} = \left(F_{\mathrm{CO,in}} - F_{\mathrm{CO,out}}\right) \cdot \mathrm{mol}_{\mathrm{CO,in}}{}^{-1} \qquad \text{(Supplementary Equation 1)}$$

$$\mathrm{CTY} = F_{\mathrm{CO,in}} \cdot X_{\mathrm{CO}} \cdot V_{\mathrm{m}}{}^{-1} \cdot m_{\mathrm{Co}}{}^{-1} \qquad \text{(Supplementary Equation 2)}$$

In Supplementary Equation 1, X_{CO} refers to the CO conversion, and $F_{\mathrm{CO,in}}$ and $F_{\mathrm{CO,out}}$ refer to flow of CO in and out of the reactor respectively. In Supplementary Equation 2, V_{m} is the molar volume and m_{Co} is the mass of cobalt loaded in the reactor.

The selectivity towards carbon dioxide is calculated as in (Supplementary Equation 3). The selectivity towards light hydrocarbon products with carbon number n (with $1 \leq n \leq 4$) is calculated as in (Supplementary Equation 4), and the selectivity towards the heavier fraction ($n \geq 5$) as in (Supplementary Equation 5).

$$S_{\mathrm{CO_2}} = F_{\mathrm{CO_2}} \cdot (F_{\mathrm{CO,in}} \cdot X_{\mathrm{CO}})^{-1} \qquad \text{(Supplementary Equation 3)}$$

$$S_{\mathrm{C1-C4}} = F_{\mathrm{C}n} \cdot n \cdot (F_{\mathrm{CO,in}} \cdot X_{\mathrm{CO}})^{-1} \qquad \text{(Supplementary Equation 4)}$$

$$S_{\mathrm{C5+}} = 1 - S_{\mathrm{C1-C4}} - S_{\mathrm{CO_2}} \qquad \text{(Supplementary Equation 5)}$$

In this case, $S_{\mathrm{C}n}$ represents the selectivity towards hydrocarbon product of a specific carbon number and $F_{\mathrm{C}n}$ is the flow of the corresponding hydrocarbon product. Likewise, S_{CO2} and F_{CO2} denote the CO_2 selectivity and flow of CO_2 out of the reactor respectively.

Supplementary References

1. Zhong, L. *et al.* Cobalt carbide nanoprisms for direct production of lower olefins from syngas. *Nature* **538,** 84–87 (2016).

2. Torres Galvis, H. M. *et al.* Supported iron nanoparticles as catalysts for sustainable production of lower olefins. *Science* **335,** 835–8 (2012).

3. Jiao, F. *et al.* Selective conversion of syngas to light olefins. *Science (80-.).* **351,** 1065–1068 (2016).

4. Vila, E., Rojas, R. M., Martín de Vidales, J. L. & García-Martínez, O. Structural and Thermal Properties of the Tetragonal Cobalt Manganese Spinels MnxCo3-xO4 (1.4 < x < 2.0). *Chem. Mater*. **8,** 1078–1083 (1996).
